# Mitochondrial Superoxide Contributes to Blood Flow and Axonal Transport Deficits in the Tg2576 Mouse Model of Alzheimer's Disease

**DOI:** 10.1371/journal.pone.0010561

**Published:** 2010-05-10

**Authors:** Cynthia A. Massaad, Samir K. Amin, Lingyun Hu, Yuan Mei, Eric Klann, Robia G. Pautler

**Affiliations:** 1 Department of Molecular Physiology and Biophysics, Baylor College of Medicine, Houston, Texas, United States of America; 2 Department of Cognitive Science, Rice University, Houston, Texas, United States of America; 3 Center for Neural Science, New York University, New York, New York, United States of America; Federal University of Rio de Janeiro, Brazil

## Abstract

**Background:**

Alzheimer's disease (AD) is a neurodegenerative disease characterized by the progressive decline in cognitive functions and the deposition of aggregated amyloid β (Aβ) into senile plaques and the protein tau into tangles. In addition, a general state of oxidation has long been known to be a major hallmark of the disease. What is not known however, are the mechanisms by which oxidative stress contributes to the pathology of AD.

**Methodology/Principal Findings:**

In the current study, we used a mouse model of AD and genetically boosted its ability to quench free radicals of specific mitochondrial origin. We found that such manipulation conferred to the AD mice protection against vascular as well as neuronal deficits that typically affect them. We also found that the vascular deficits are improved via antioxidant modulation of the endothelial nitric oxide synthase, an enzyme primarily responsible for the production of nitric oxide, while neuronal deficits are improved via modulation of the phosphorylation status of the protein tau, which is a neuronal cytoskeletal stabilizer.

**Conclusions/Significance:**

These findings directly link free radicals of specific mitochondrial origin to AD-associated vascular and neuronal pathology.

## Introduction

Alzheimer's disease (AD) is a progressive neurodegenerative disease characterized by two main pathologic hallmarks: 1) extracellular senile plaques primarily comprised of accumulated amyloid β (Aβ) peptide and 2) intracellular neurofibrillary tangles comprised mainly of aggregated hyperphosphorylated tau [1], [2]. A large body of evidence has demonstrated that pathologically altered amyloid precursor protein (APP) processing is a central player in the AD etiology [1]. Additionally, there is significant evidence implicating oxidative stress as a key proponent in the events leading to AD [3]. High reactive oxygen species (ROS) levels are typically associated with the aging process, where there is ROS overproduction in conjunction with a reduction in the cellular antioxidant defense [4]. Most cases of AD present with signs of oxidative damage including lipid peroxidation, protein carbonylation, and DNA damage [5], [6], [7]. In addition, aspects of Aβ toxicity have been attributed to its oxidative capability. Indeed, Aβ has been shown to promote oxidation in several model systems [8], [9], increase hydrogen peroxide levels, and decrease cytochrome C oxidase activity in the Tg2576 mouse model of AD [10]. Aβ has also been shown to induce the formation of ROS in mature hippocampal neurons, mediated via N-methyl-D-Aspartate (NMDA) receptors. This effect was blocked by a mitochondrial uncoupler, indicating that the oxidative stress is of mitochondrial origin [11]. Additionally, Aβ has been shown to enter mitochondria and compromise their integrity through inactivation of the superoxide dismutase 2 (SOD-2) and a subsequent increase in superoxide levels [12]. Aβ-induced mitochondrial dysfunction has further been linked to AD through studies showing that the genetic reduction of SOD-2 in AD model mice can amplify multiple AD symptoms [13], [14] and lead to increased plaque deposition [15]. Conversely SOD-2 overexpression can alleviate several AD-related symptoms, most notably the learning and memory deficits characteristic of AD [16], [17].

There is clearly a prominent role for mitochondrial superoxide in mediating the effects of Aβ on neuronal function [16]. Aβ, however, not only accumulates in neuronal parenchyma, but can also deposit on blood vessel walls in a process referred to as cerebral amyloid angiopathy (CAA) [18], [19]. CAA has been documented extensively in AD, with a wealth of evidence linking AD with vascular dysfunction. This evidence includes existence of cerebrovascular disease in the AD brain, blood brain barrier dysfunction, and several common predisposing cerebrovascular risk factors, such as stroke, heart disease, hypertension and atherosclerosis [20], [21], [22], [23]. The endothelial nitric oxide synthase (eNOS) produces nitric oxide (NO) [24], which is responsible for smooth muscle relaxation and regulation of muscle tone [25], [26], [27]. During conditions of oxidative stress, production of superoxide and its derived oxidants, induce uncoupling of eNOS leading to the production of NOS-derived superoxide instead of NO [28], [29]. This effect is mediated primarily by Akt-dependent phosphorylation of eNOS at serine 1177 [30], [31]. The subsequent imbalance between NO and superoxide potentiates oxidative stress and can contribute to the onset of a variety of vascular diseases in multiple organs [32], including the brain which is particularly vulnerable to oxidative damage [33].

Although ROS have been well documented to contribute to both vascular and neurodegenerative diseases, the specific source of ROS for each particular disease is still unresolved. Given the solid link between mitochondrial dysfunction and AD, our interest lies in elucidating the effects of mitochondrial ROS on AD pathology. We have already demonstrated the involvement of mitochondrial superoxide in the learning and memory deficits characteristic of AD [16]. Our next step was to identify whether this effect extended systemically to the vascular system. Multiple studies implicate mitochondrial ROS with vascular and neuronal dysfunction. For example, using mutant mice with reduced expression of SOD-2, Wenzel and colleagues demonstrated an increased oxidative stress with disrupted vascular function in these animals in relation to cardiac disease [34]. Iadecola and colleagues used an NADPH oxidase mutant mouse and showed similar effect of ROS on the brain vasculature [35].

Vascular homeostasis is of paramount importance for proper neuronal function, as blood flow is required to maintain normal neuronal physiology. AD-related blood flow deficits have been correlated with neuronal dysfunction characteristic of the disease [36], [37]. Of particular interest is the disruption of axonal transport during AD [38]. Axonal transport relies amongst other on a network of cytoskeletal proteins and their associated molecules. One such microtubule-associated protein is tau, of which the involvement in the pathology of AD has been extensively documented. Tau functions as a microtubule stabilizing protein and its partial phosphorylation is required for its normal function. Tau hyperphosporylation, however, leads to two negative consequences: 1) loss of function by dislodging from microtubules and subsequently destabilizing them and 2) gain of toxic function by its capacity to sequester normal tau and other microtubule associated proteins. Therefore there is little doubt that abnormal tau metabolism contributes to decreased axonal transport associated with AD [39]. Aβ and oxidative stress have also been suggested to contribute to the axonal pathology observed in AD [40]. Less clear, however, is the mechanism by which this contribution occurs.

AD-related vascular, mitochondrial, and neuronal dysfunctions collectively led us to the formulation of our hypothesis that mitochondrial superoxide is a central player in the mechanisms by which Aβ instills multiple AD pathologies, including vascular and neuronal dysfunction. Therefore, we posited that SOD-2 overexpression could prevent AD-related blood flow impairments and axonal transport deficits in the Tg2576 mouse model of AD. To test this hypothesis, we utilized magnetic resonance imaging (MRI) to assess cerebral blood flow and the rates of axonal transport in the Tg2576 mice overexpressing SOD-2 at ages ranging from 4 to 16 months. Levels of eNOS and tau as well as their modulation by phosphorylation were also determined.

Our findings suggest that Aβ can exert widespread effects partially via mitochondrial ROS impacting multiple systems, most notably vascular and neuronal physiology. This impact may be a key event in the etiology of AD-related blood flow and axonal transport deficits and helps elucidate the cellular mechanisms that precipitate neuronal degeneration. The understanding of such events is crucial to preventing or potentially reducing neuronal injury during AD.

## Results

### The effect of mitochondrial superoxide on the cerebral blood flow in AD

**Figure 1:**
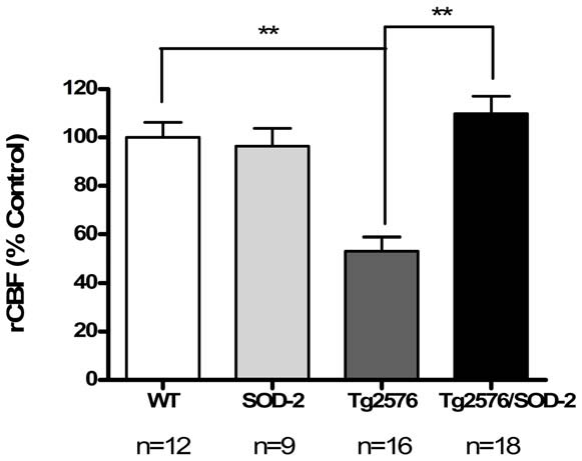
SOD-2 overexpression improves the regional cerebral blood flow (rCBF) deficits displayed in Tg2576 mice. **A**) The graph represents the regional cerebral blood flow (rCBF) levels in 12 to 16 months old WT, SOD-2, Tg2576 and Tg2576/SOD-2 mice as measured by MRI (ASL). Significance was assessed by a one-way ANOVA with Dunnett's post-test for multiple comparisons. ** p<0.01.

Utilizing the MRI methodology arterial spin labeling (ASL), we measured the regional cerebral blood flow (rCBF) in the cortex of Tg2576 mice with or without SOD-2 overexpression, at ages ranging from 4 to 16 months. We found that aged Tg2576 mice (12 to 16 months of age) exhibited a severe blood flow deficit compared to age-matched WT littermate controls (overall p<0.0001) (Figure 1). Overexpression of SOD-2, and therefore reduction of mitochondrial superoxide, resulted in a recovery of the rCBF back to normal levels (Figure 1). Similar studies at 8 months of age, when levels of amyloid β have already begun to increase but prior to plaque depostion, revealed the same pattern of blood flow deficits in Tg2576 mice that was recovered upon overexpression of SOD-2 (overall p = 0.0139) (Figure S1). At 4 months of age when amyloid β pathology has not presented yet, none of the mice studied showed a significant blood flow deficit (Figure S1). Interestingly, SOD-2 mice exhibited a slightly lower blood flow rate without reaching significance. This is consistent with the notion that at a younger age, superoxide is a necessary molecule, and therefore quenching it may result in adverse effects.

### The effect of mitochondrial superoxide on eNOS function in AD

**Figure 2:**
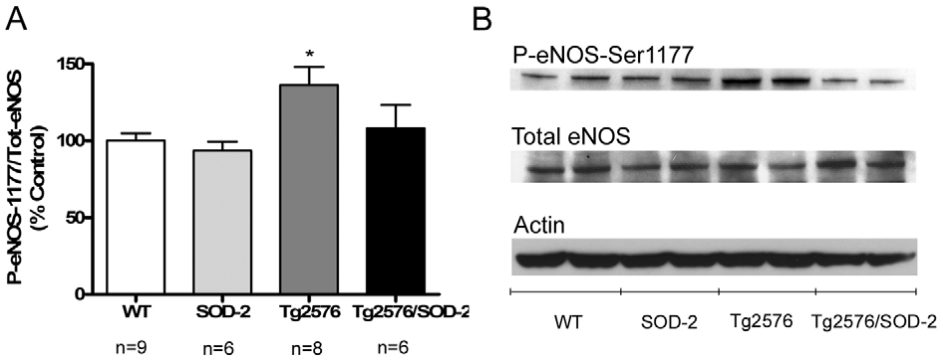
SOD-2 overexpression prevents increases in phospho-eNOS-ser1177 in Tg2576 mice. **A**) Graph represents quantification of the levels of phospho-eNOS-ser1177 normalized to total eNOS from 12 to 16 months old cortical homogenates of WT, SOD-2, Tg2576 and Tg2576/SOD-2 mice. Significance was assessed by one way ANOVA with Dunnett's post-test for multiple comparisons. *p<0.05. **B**) Representative Western blot of phospho-eNOS-Ser1177, total eNOS and β-actin from cortical homogenates of 12 to 16 months old WT, SOD-2, Tg2576 and Tg2576/SOD-2 mice.

A possible mechanism by which mitochondrial superoxide could modulate blood flow is through modulation of eNOS function. We therefore measured the levels of phospho-eNOS (ser1177) in cortical lysates from old mice (12 to 16 months of age). Because the amount of eNOS protein in the sample could potentially contribute to the amount of phosphorylation at serine 1177, we normalized phospho-eNOS-Ser1177 levels to the amount of total eNOS protein. Also to account for variability in loading, we measured the levels of β-actin in the same samples and found them to be unchanged. Our data indicated that the levels of phospho-eNOS in cortical fractions were significantly increased in the Tg2576 mice (overall p = 0.021) (Figure 2), which correlated with the blood flow deficits observed in the same mice. Moreover, overexpression of SOD-2 in the Tg2576 mice reduced the levels of phospho-eNOS back to those in wild-type mice (Figure 2), which corresponded with the recovered blood flow deficits in these same mice.

### The effect of mitochondrial superoxide on axonal transport rates in AD

**Figure 3:**
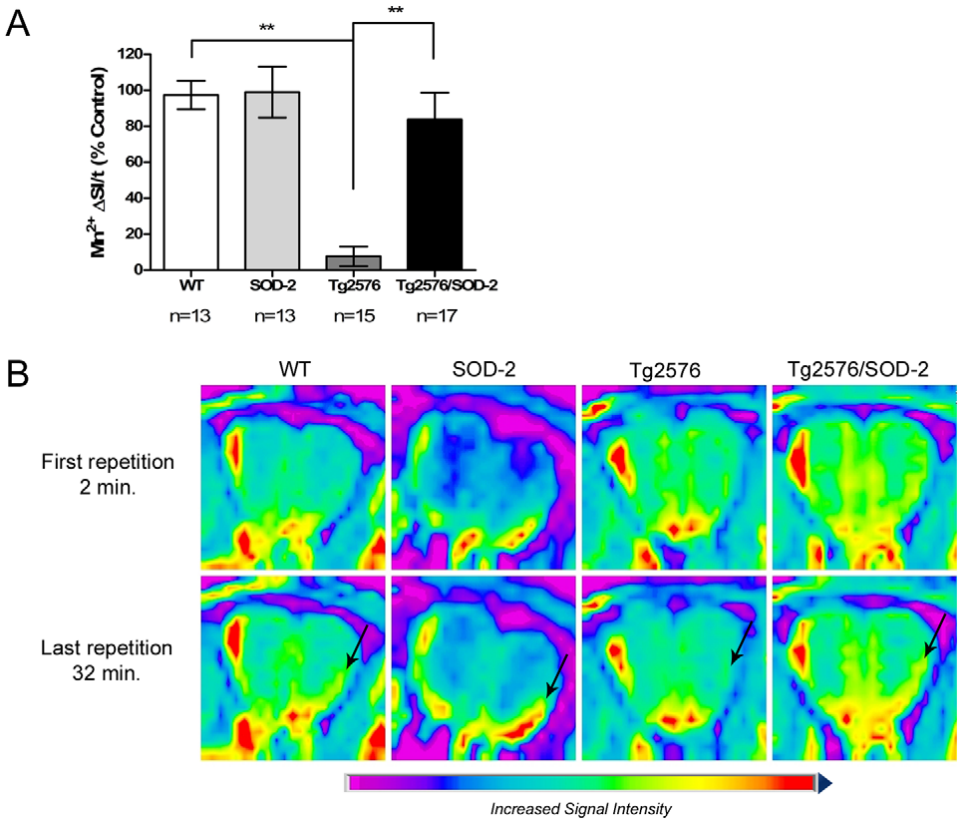
SOD-2 overexpression improves the axonal transport deficits displayed by Tg2576 mice. **A**) The graph represents the rates on axonal transport as measured *in vivo* by MEMRI in 12 to 16 months old WT, SOD-2, Tg2576 and Tg2576/SOD-2 mice. Significance was assessed by a one-way ANOVA with Dunnett's post-test for multiple comparisons. ** p<0.01. **B**) Representative MR images with a pseudo-color overlay showing manganese accumulation in the region of interest (indicated by an arrow) in the olfactory bulb of 12 to 16 months old WT, SOD-2, Tg2576 and Tg2576/SOD-2 mice. Top row images represent the first repetition of the scan, where axonal transport of manganese has not started yet. Bottom row images represent the last repetition of the scan, where most of the manganese has already accumulated in the region of interest. Note that for the Tg2576 mice, no manganese accumulated in the region of interest which is indicative of deficient axonal transport. In the Tg2576/SOD-2 mice, accumulation of manganese is intact, indicating recovered axonal transport. The two images from each animal are on the same brightness scale.

Using a novel dynamic MRI imaging technique, MEMRI, we measured *in vivo* axonal transport rates in the Tg2576 mice, with or without SOD-2 overexpression, at ages ranging from 4 to 16 months. We found that aged Tg2576 mice (12 to 16 months of age) exhibited a severe impairment in axonal transport rates compared to the age-matched WT littermate controls (overall p<0.0001) (Figure 3). This deficit was rescued with overexpression of SOD-2, indicating that mitochondrial superoxide plays a key role in the axonopathy associated with AD. A similar pattern was observed at 8 months of age. In this age group, however, the axonal transport rates deficits were partially rescued (overall p<0.0001) (Figure S2). The youngest group of mice (4 months of age) did not exhibit any axonal transport deficits, regardless of the genotype studied (Figure S2).

### The effect of mitochondrial superoxide on tau hyperphosphorylation in AD

**Figure 4:**
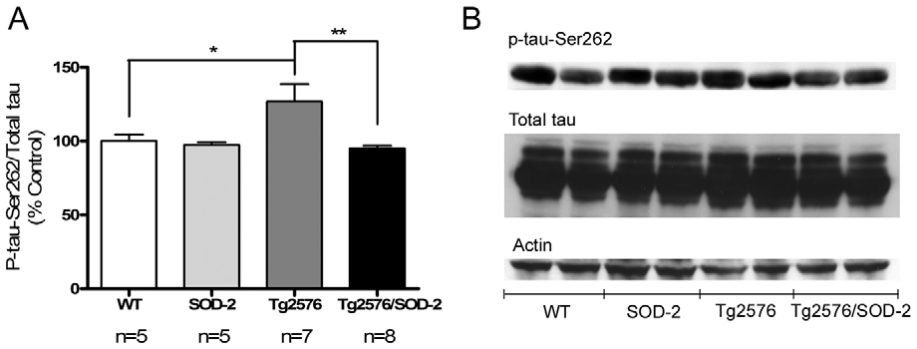
SOD-2 overexpression prevents increases in phospho-tau-ser262 in Tg2576 mice. **A**) Graph represents quantification of the levels of phospho-tau-ser262 normalized to total tau from 12 to 16 months old olfactory bulb homogenates of WT, SOD-2, Tg2576 and Tg2576/SOD-2 mice. Significance was assessed by one way ANOVA with Dunnett's post-test for multiple comparisons. *p<0.05; **p<0.01. **B**) Representative Western blot of phospho-tau-Ser262, total tau and β-actin from olfactory bulb homogenates of 12 to 16 months old WT, SOD-2, Tg2576 and Tg2576/SOD-2 mice.

During AD, axonal transport rate dysfunction has been in part associated with microtubule destabilization, following hyperphosphorylation of the microtubule-associated protein tau [41]. We measured the levels of phospho-tau at multiple epitopes (199, 202, 205, 231 and 356) but could not detect hyperphosphorylation of tau at these sites in Tg2576 mice (data not shown). We detected tau hyperphosphorylation at the serine 262 site (located in the microtubule binding domain of tau), in olfactory bulb homogenates of aged mice (12 to 16 months). Because the amount of tau in the sample could potentially contribute to the amount of phosphorylation at serine 262, we normalized phospho-tau levels to the amount of total tau protein. Also to account for variability in loading, we measured the levels of β-actin in the same samples and found them to be unchanged. We observed a significant increase in the levels of phospho-tau (ser262) in the olfactory bulbs of Tg2576 mice (overall p = 0.0099) (Figure 4). This increase was recovered back to wildtype levels upon SOD-2 overexpression (Figure 4), coinciding with the recovery from axonal transport deficits in these animals.

## Discussion

Herein we have shown that reducing mitochondrial superoxide by SOD-2 overexpression ameliorated cerebral blood flow deficits and axonal transport deficits typically exhibited by Tg2576 mice. The reduction of mitochondrial superoxide also resulted in a concomitant reduction of phosphorylation of eNOS at serine 1177, as well as phosphorylation of tau at serine 262. Utilizing the same mouse model, we have previously shown that SOD-2 overexpression did not alter the elevated levels of Aβ during AD but resulted in a decrease in the Aβ42/Aβ40 ratio, indicative of an Aβ pool less favorable for aggregation. SOD-2 is the main superoxide scavenger in mitochondria. Therefore, we interpret our data as direct *in vivo* evidence that mitochondrial superoxide is a key player in AD-related vascular and neuronal dysfunction, working as a downstream effector of Aβ, possibly affecting Aβ processing.

### Aβ, blood flow and eNOS

One of the earliest events in the development of AD pathology is a progressive elevation of Aβ which eventually is secreted and accumulates as senile plaques, not only on brain parenchyma but also within the vasculature [1], [42]. In the Tg2576 mouse model of AD, Aβ has been shown to deposit within the vasculature beginning at approximately 9 months of age and continuing as aging ensues [42]. Aβ vascular deposition has been linked to vascular dysfunction, specifically blood flow deficits, occurring during the development of AD [37]. In this particular mouse model of AD, soluble Aβ levels are elevated beginning at 6 months of age, well before vascular Aβ deposition [42]. Similar to the wealth of studies implicating soluble Aβ in AD-related toxicity, it has been suggested that soluble Aβ plays a role in the blood flow deficits as well, possibly contributing to the early progression of AD via a systemic effect on the vasculature. Based on this hypothesis, Shin and associates measured cerebral blood flow in 8 month- and 18 month-old Tg2576 mice using laser speckle flowmetry through an intact skull [37]. They confirmed the blood flow deficits in the aged group, but failed to record any differences in cerebral blood flow and vascular physiology between 8 month-old Tg2576 mice and their wild-type littermate controls [37]. In our studies, we utilized a non-invasive MRI technique to measure cerebral blood flow in Tg2576 mice and confirmed that it is significantly deficient post vascular plaque deposition (12–16 months of age). However, in contrast to the previous study, we were able to detect a significant deficiency in cerebral blood flow at 8 months of age, prior to Aβ vascular deposition. This discrepancy could be due to the differences in imaging modalities utilized, as well as to the different regions of interest analyzed; we have previously noted differences within the Tg2576 mice depending upon where the images were acquired (data not shown). We also showed that reducing mitochondrial superoxide by SOD-2 overexpression led to improvement of the blood flow deficits in Tg2576 mice at both age points, indicating that mitochondrial superoxide exerts a global impact on the vascular system, early on during soluble Aβ elevation. The debate remains whether excess mitochondrial superoxide contributes to Aβ elevation or whether Aβ elevation occurs first and causes an increase in superoxide levels. We and others recently performed behavioral and biochemical studies using a similar animal model and demonstrated that SOD-2 overexpression improved AD-related learning and memory deficits without reducing the elevated levels of Aβ [16], [17]. These studies suggest that mitochondrial superoxide exerts its effect downstream of Aβ. In our previous study, however, we demonstrated that SOD-2 overexpression causes a reduction in the Aβ42/Aβ40 ratio, thereby modifying the Aβ pool towards a less amyloidogenic composition [16]. The latter suggests that mitochondrial superoxide can also affect Aβ processing. Additionally, Guglielmotto and associates demonstrated that brain hypoperfusion can lead to increased β-secretase activity (and therefore increased Aβ levels) via hypoxia-induced ROS overproduction from mitochondria [43]. Together these studies illustrate an amplification loop through which Aβ and mitochondrial superoxide potentiate each other's pathological effect on vascular and brain function during AD.

We also demonstrated that Tg2576 mice exhibit an increased phosphorylation of eNOS at serine 1177. eNOS expression has been shown to be decreased in the brains of AD patients [44]. Its activity, however, has not yet been measured. eNOS activity is regulated mainly via phosphorylation, with two most prominent regulatory sites: threonine 495, which is inhibitory [45] and serine 1177, which is permissive [46]. Our data therefore supports increased activation of eNOS in the aged (12–16 months old) Tg2576 mice. Most importantly, eNOS phosphorylation at serine 1177 has been shown to promote increased superoxide production from the enzyme [28], thereby contributing to the existing oxidative stress in these animals. Increased superoxide production from eNOS also means that nitric oxide production is compromised. This imbalance between nitric oxide and superoxide levels may be causative of the observed blood flow deficits, as suggested from previous studies [32]. In support of this possibility, SOD-2 overexpression returns the eNOS phosphorylation status back to normal. eNOS phosphorylation at serine 1177 is mediated by Akt [31], which has been extensively shown to be modulated by oxidative stress. Our data support previous reports and also specifically implicate mitochondrial superoxide in Akt-dependent eNOS phosphorylation at serine 1177. Collectively, these studies suggest that mitochondrial superoxide plays a key role in the initiating events of the AD-related vascular deficits.

### Aβ, axonal transport and tau

We have previously shown that the Tg2576 mouse model of AD exhibits severe deficits in axonal transport rates beginning at 7 months of age and progressively worsening in parallel to the age- and AD-related increases in Aβ levels [47]. We chose to perform our experiments in the olfactory system because it is one of the first systems affected during AD, APP/Aβ pathology occurs in the olfactory bulb [47], and because of the ease and non-invasive possibility of manganese introduction via a nasal lavage. In the current study, we confirmed the early occurrence of axonal transport rates deficits in the 8-month old Tg2576 mice as well as the maintenance of these deficits in mice12 to 16 months of age. We further extended the use of this MEMRI technique to assess whether SOD-2 overexpression would alleviate these symptoms. We found that the reduction of mitochondrial superoxide significantly prevented the deficits in axonal transport rates in the Tg2576 mice, indicating that ROS of mitochondrial origin are a key factor in transducing the impact of Aβ on neuronal physiology.

Given that SOD-2 functions with manganese as an essential cofactor, it is conceivable that SOD-2 transgenic mice may exhibit baseline positive contrast enhancement in T_1_-weighted MRI images. To ensure that this was not a confounding factor in the interpretation of our data, we measured the T_1_ and T_2_ values in 2 month-old wild-type, SOD-2, Tg2576, and Tg2576/SOD-2 mice at baseline, in the cortex, hippocampus and olfactory bulb. Our data showed no significant differences in T_1_ and T_2_ values regardless of the genotype studied (Figure S3) indicating that endogenous manganese does not significantly contribute to signal enhancement. Additionally, our MEMRI experiments were designed to measure axonal transport rates as opposed to absolute transport. Therefore, even in the event of a baseline signal enhancement in SOD-2 mice, the rate of change of signal intensity, which is representative of axonal transport rates, should not be affected.

One pathway that mitochondrial ROS could affect to alter axonal transport is through the microtubule-associated protein tau. Tau stabilizes microtubules through binding and is essential to the integrity of axonal transport [48]. Reducing endogenous levels of total tau has been shown to ameliorate the Aβ-induced pathology [49], indicating that it plays a role downstream of Aβ during the events leading to AD. Another theory about the involvement of tau in AD pertains to its phosphorylation status. During the progression of AD, there is extensive hyperphosphorylation of tau that causes it to detach from the microtubule network. This leads to the destabilization of microtubules resulting in axonal transport deficits [50]. Hyperphosporylation of tau also has been shown to contribute to neuronal death [51]. We specifically found an increase in the phosphorylation of tau at serine 262 in olfactory bulb homogenates of Tg2576 mice. This phosphorylation site is located within the microtubule binding domain of tau. Phosphorylation of tau is returned back to normal with SOD-2 overexpression, indicating that mitochondrial superoxide also affects axonal transport through tau dysfunction. Additionally, to extend the pathophysiological significance of such results to areas of the brain that are more directly relevant to cognitive deficits observed in AD, we measured the levels of phosphorylated tau at serine 262 in cortical homogenates of aged mice, which include frontal cortex, hippocampus and amygdala. We observed a significant increase in the levels of phopshorylated tau (serine 262) in Tg2576 mice compared to controls (overall p<0.001) (Figure S4). This increase was recovered back to wildtype levels upon SOD-2 overexpression, suggesting that mitochondrial superoxide contributes to tau pathology not only in the olfactory bulb, but also extends to the learning and memory centers of the brain. It is not clear, however, what are the mechanisms of such an effect. An attractive possibility can be related to the blood flow deficits in the first portion of this study. We have demonstrated an effect of mitochondrial ROS on blood flow which is known to affect glucose metabolism. According to this scheme, decreased blood flow lead to a decrease in glucose metabolism, and particularly a reduction in the sugar O-GlcNAC. O-GlcNACylation is a well documented post-translational modification of tau that competes for the same sites as its phosphorylation [52]. Therefore, decreased O-GlcNACylation of tau results in its increased phosphorylation due to lack of binding competition [52]. Hyperphosphorylation of tau in turns results in decreased axonal transport rates. Therefore, it is possible that mitochondrial ROS affect nerve function via a global effect on the vasculature. This is not uncommon, as blood flow is known to be required for the integrity of neuronal function. An alternative explanation is that mitochondrial ROS exert a direct effect on the signaling pathway leading to hyperphosphorylation of tau. For example, the glycogen synthase kinase 3 beta (GSK-3β), one of the major kinases modulating tau phosphorylation has been shown to be inhibited by antioxidant treatment [53], indicating that ROS activate the enzyme and result in increased phosphorylation of tau. Further experiments are required to clarify whether ROS affect tau phosphorylation directly or via their effect on blood flow. Regardless of the mechanism however, it is clear that mitochondrial superoxide plays a central role in neuronal pathology as exemplified by axonal transport deficits.

### Concluding remarks

Although oxidative stress has long been known to contribute to AD pathology both in its early and late stages, the mechanisms of such contribution remain unclear. One hypothesis implicates Aβ as the initiator of AD. Aβ is a powerful oxidant and therefore, as its levels are progressively elevated during the progression of the disease, so are the levels of ROS. On the other hand, with the exception of the genetic cases, sporadic AD is a disease of the elderly and oxidative stress is known to occur during physiological aging in the absence of elevated Aβ levels. Therefore, oxidative stress could also be contributing to the initiating factors of AD. We would like to emphasize the important issue of the production source of ROS. Although oxidative stress has been shown to contribute to the pathology of aging, we have specifically shown that mitochondrial ROS do not participate in synaptic plasticity and memory impairments occurring in aged mice [54]. One possible reason is the selective nature of the inner mitochondrial membrane which does not allow superoxide leakage. During AD, mitochondrial dysfunction is very well documented and thus, it is possible that mitochondrial superoxide leaks across the mitochondrial membrane. The vascular endothelium and the brain both have high energy demands and contain high levels of mitochondria, and are therefore more vulnerable to mitochondrial ROS, especially when their levels go out of balance. Although various antioxidant therapies have been attempted to treat AD, they were only mildly effective, perhaps because of their non-specific nature. Our data provides strong evidence that mitochondrial-derived superoxide acts possibly as a mediator and/or potentiator of the toxic effects of Aβ systemically on the vasculature and regionally on nerve physiological functioning. Therefore, mitochondria are an excellent target candidate for antioxidant drug development to treat AD.

## Methods

### 1. Transgenic Mouse Lines

We crossed male Tg2576 AD mice with SOD-2 overexpressing females and obtained progeny of WT, SOD-2, Tg2576 and Tg2576/SOD-2 mice. Both parent transgenic lines have been previously described [55], [56]. Briefly, the Tg2576 mouse carries the Swedish mutant human amyloid precursor protein (APP) under the hamster prion promoter and the SOD-2 transgenic mouse overexpresses the mitochondrial superoxide dismutase under the β-actin promoter. Experiments were performed at 4, 8, and 12–16 months of age. Genotypes were determined by PCR using tail DNA with the following primers: 5′-CTG-ACC-ACT-CGA-CCA-GGT-TCT-GGG-T-3′ and 5′-GTG-GAT-AAC-CCC-TCCCCC-AGC-CTA-GAC-CA-3′ for APP; and 5′-CAC-AAG-CAC-AGC-CTC-CCA-G-3′ and 5′-CGC-GTT-AAT-GTG-TGG-CTC-C-3′ for SOD-2. All mice were housed at Baylor College of Medicine's transgenic mouse facility in compliance with the National Institutes of Health guidelines for Care and Use of Laboratory Animals. The mouse facility is kept on a 12-hour light–dark cycle, with a regular feeding and cage-cleaning schedule. All experiments were conducted and approved by Baylor College of Medicine's IACUC.

### 2. MRI Studies

#### A. Animal preparation

All mice were initially anesthetized with 5% isoflurane in oxygen. Following transfer to the animal holder within the magnet, anesthesia was maintained at 1.5–2% isoflurane in oxygen. The animal's temperature was held constant at 37°C using an air heating system and monitored with a rectal probe (Small Animal Instruments (SAI), Inc. (Stony Brook, NY)). Respiratory rates were also monitored utilizing a respiratory probe and monitored during the entire imaging session ((Small Animal Instruments (SAI), Inc. (Stony Brook, NY)). All imaging protocols were conducted utilizing a 9.4 T, Bruker Avance Biospec Spectrometer, 21 cm bore horizontal imaging system with a 35 mm volume resonator (Bruker BioSpin, Billerica, MA).

#### B. *In vivo* axonal transport measurements

We measured axonal transport rates *in vivo* utilizing a Manganese Enhanced MRI (MEMRI) protocol as we have described previously [47]. Briefly, a concentrated solution of MnCl_2_ (0.77 g/ml) was pipetted into the nasal cavity of the mouse at a total of 4 µl (2 µl/naris). Mice were allowed to recover for 45 minutes on a heating pad, allowing the loading of Mn^2+^ into the olfactory receptor neurons located in the olfactory epithelium. The mice were then sedated with isoflurane and loaded into the magnet. The zero time point for imaging was at 60 minutes post Mn^2+^ exposure. Spin lattice (T_1_)-weighted, spin-echo 2D data sets were acquired of the mouse brain using a multi-slice/multi-echo 2D imaging protocol with the following parameters: matrix dimensions  = 128×128; FOV = 3.0 cm×3.0; slice thickness = 1 mm; repetition time (TR) = 504.1 ms; echo time (TE)  = 8.2 ms; number of averages (NA)  = 2; number of repetitions = 15; time per image = 2 min. Four axial slices were selected with the first slice aligned with the leading edge of the olfactory bulb. In all studies, slice 2 of the 4 slices was assayed for axonal transport in a region of interest (ROI) selected in the dorsal lateral portion of the olfactory bulb. Changes in the signal intensity of this ROI were measured using Bruker's Paravision software and plotted using Microsoft Excel and GraphPad Prism (GraphPad Software, Inc., (LaJolla, CA)). All signal intensities were normalized to non-enhanced muscle outside of the brain. A least squares method was used to determine the change in signal intensity over time, reflective of the rate of transport of Mn^2+^.

#### C. Cerebral blood flow measurements

We measured the regional cerebral blood flow (rCBF) with the MRI imaging protocol, arterial spin labeling (ASL). We utilized a flow alternating inversion recovery-echo planar imaging (FAIR-EPI) protocol with the following imaging parameters: matrix dimensions = 64×64; FOV = 1.5 cm×1.5 cm; slice thickness = 1.5 mm; TR = 7555.377 ms; TE = 16.73 ms; NA = 1; number of repetitions = 1; flip angle = 90; inversion recovery time (TIR)  = 18.25 ms; number of TIR values = 16; increment of TIR = 500 ms; inversion slab thickness (for the selective inversion)  = 5 mm. In order to determine the proper reproducible section location, a standard scan of the whole brain was obtained with 16 coronal slices spanning from the leading edge of the olfactory bulb to the trailing edge of the cerebellum. The slice of interest was determined as the first coronal section where the rostral portion of the hippocampus appears. The location of this particular slice was noted and used in the blood flow protocol. The ROI was manually selected in the entire cortex region spanning the width of the hippocampus in the slice of interest. T_1_ values from the scans with selective and non-selective inversions were calculated using the t1invacq function in Bruker's paravision 4.0. Blood flow was calculated using the following formula: rCBF = λ(1/T_1 selective_−1/T_1 non-selective_) where λ is the blood brain partition coefficient, equivalent to 0.9 (or 90 g per 100 g) for the mouse brain. Data was plotted using GraphPad Prism (GraphPad software, Inc. (La Jolla, CA)).

#### D. T_1_ and T_2_ measurements

We measured the T_1_ and spin-spin relaxation rates (T_2_) values in 2 months old WT, SOD-2, Tg2576 and Tg2576/SOD-2 animals at baseline, in the cortex, hippocampus and olfactory bulb. In order to determine the proper reproducible section location, a standard scan of the whole brain was obtained with 16 coronal slices spanning from the leading edge of the olfactory bulb to the trailing edge of the cerebellum. The slice of interest for the cortex/hippocampus was determined as the first coronal section where the rostral portion of the hippocampus appears, matching the section chosen for the rCBF studies. The slice of interest for the olfactory bulb was chosen to match the slice selected for the axonal transport studies. To acquire the T_1_ values, the brain was imaged using a 2D spin echo sequence with a dynamic TR parameter: TR  = 326.38, 928.309, 1949.379, 7500 ms. Other parameters included TE  = 6.6 ms, FOV = 2.5 cm, slice thickness = 1 mm, NA = 1, matrix = 128×128. T_2_ weighted images were acquired using the following settings: TR = 1000 ms, TE = 10.3, 20.6 ms, FOV = 3.0 cm, slice thickness = 1 mm, NA = 1, matrix = 128×128. The olfactory bulb was imaged using the same parameters. Analysis of MR images was conducted using Bruker's paravision 4.0. For the cortex and hippocampus, the ROIs were drawn manually in each hemisphere. For the olfactory bulb, only the right side was used, due to the presence of the chemical shift artifact on the left side. ROIs for the cortex and olfactory bulb matched the ones selected in the CBF and MEMRI studies, respectively. The ROI for the hippocampus covered the entire surface of the rostral hippocampus, in the slice selected, as described above. The T_1_ recovery and T_2_ decay values were calculated for each brain region and plotted using GraphPad Prism (GraphPad software, Inc. (La Jolla, CA)).

### 3. Brain Sample Preparation

Mice were sacrificed by cervical dislocation then their brains were extracted fresh on ice. The left hemispheres were further dissected to obtain the olfactory bulb and cortex. The olfactory bulb was homogenized by sonication in 250 µl of Tris buffered saline (TBS) containing protease and phosphatase inhibitors and the cortex was homogenized with a glass dounce homogenizer in 2 ml of the same buffer (137 mM NaCl, 20 mM Tris, pH 7.6, 1∶100 of each of protease inhibitors cocktail, phosphatase inhibitor cocktail I and phosphatase inhibitor cocktail II (Sigma-Aldrich)). Homogenates were centrifuged at 5000 g for 5 minutes, and then the supernatants were aliquoted and saved at −80°C until further use.

### 4. Immunoblotting

Total protein from brain samples were measured with the DC assay (Bio-Rad Laboratories (Hercules, CA)), and equally loaded and separated by SDS/PAGE. Protein samples were then transferred onto a nitrocellulose membrane that was blocked with a TBS solution containing 2.5% w:v milk, 2.5% w:v bovine serum albumin, 0.1% Tween 20 and 0.05M NaF. The membranes were then probed with 1∶250 anti-phospho-262-tau antibody (Santa Cruz Biotechnology, Inc. (Santa Cruz, CA)), 1∶5000 anti-tau (Dako North America, Inc. (Carpinteria, CA)), 1∶1000 anti-phospho-eNOS-1177 (Cell Signaling Technology, Inc. (Danvers, MA)), 1∶1000 anti-eNOS (Cell Signaling Technology, Inc. (Danvers, MA)) or 1∶10000 anti-β-actin (Sigma-Aldrich (St. Louis, MO)). This initial incubation was then followed by a horseradish peroxidase-conjugated secondary antibody (Promega Corporation (Madison, WI)) against mouse IgG (for actin) and against rabbit IgG (for all remaining). Development was achieved by autoradiography in a Kodak X-omat 2000A processor using ECL (GE Healthcare Life Sciences (Piscataway, NJ)) or Super Signal (Thermo Fisher Scientific (Rockford, IL)) as needed.

For band analysis, films were scanned using an EPSON scanner and then band density calculated using Image J 1.38x (National Institutes of Health, USA). Data was plotted using GraphPad prism (GraphPad Software, Inc. (La Jolla, CA)).

### 5. Statistics

In all experiments mentioned above, statistical analyses were carried out in Prism (GraphPad Software, Inc. (La Jolla, CA)). Statistical significance was calculated using a one way analysis of variance (ANOVA) followed by Dunnett's post-test for multiple comparisons. A p-value of 0.05 or less was considered statistically significant.

## Supporting Information

Figure S1 Regional cerebral blood flow measurements at 4 and 8 months of age. A) The graph represents the cerebral blood flow levels in 4 months old WT, SOD-2, Tg2576 and Tg2576/SOD-2 mice as measured by MRI (ASL). No differences in blood flow levels between the different genotypes are observed. B) The graph represents the cerebral blood flow levels in 8 months old WT, SOD-2, Tg2576 and Tg2576/SOD-2 mice as measured by MRI (ASL). Tg2576 mice exhibit a significant deficit in their blood flow that is recovered by SOD-2 overexpression. Significance was assessed by a one-way ANOVA with Dunnett's post-test for multiple comparisons. **p<0.01.
(0.87 MB TIF)

Figure S2 Axonal transport rates measurements at 4 and 8 months of age. A) The graph represents the axonal transport rates measured *in vivo* by MEMRI in 4 months old WT, SOD-2, Tg2576 and Tg2576/SOD-2 mice. No differences in axonal transport rates between the different genotypes are observed. B) The graph represents the axonal transport rates measured *in vivo* by MEMRI in 8 months old WT, SOD-2, Tg2576 and Tg2576/SOD-2 mice. Tg2576 mice exhibit a significant deficit in their axonal transport rates that is at least partially recovered by SOD-2 overexpression. Significance was assessed by a one-way ANOVA with Dunnett's post-test for multiple comparisons. **p<0.01.
(0.85 MB TIF)

Figure S3 SOD-2 overexpression and the APP-Swedish mutation do not affect the T1 recovery and T2 decay times. A-C) Graphs represent the T1 recovery times measured in the cortex, hippocampus and olfactory bulb of 2 months old WT, SOD-2, Tg2576 and Tg2576/SOD-2 mice. D-F) Graphs represent the T2 decay measured in the cortex, hippocampus and olfactory bulb of 2 month old WT, SOD-2, Tg2576 and Tg2576/SOD-2 mice.
(1.35 MB TIF)

Figure S4 SOD-2 overexpression prevents increases in phospho-tau-ser262 in the brain of Tg2576 mice. A) Graph represents quantification of the levels of phospho-tau-ser262 normalized to total tau from 12 to 16 month old brain homogenates of WT, SOD-2, Tg2576 and Tg2576/SOD-2 mice. Significance was assessed by one way ANOVA with Dunnett's post-test for multiple comparisons. **p<0.01. B) Representative Western blot of phospho-tau-Ser262, total tau and β-actin from brain homogenates of 12 to 16 month old WT, SOD-2, Tg2576 and Tg2576/SOD-2 mice.
(0.37 MB TIF)
