# Supplementary figures and images for: Mitochondrial Superoxide Contributes to Blood Flow and Axonal Transport Deficits in the Tg2576 Mouse Model of Alzheimer's Disease
Source: PLoS One. 2010 May 10;5(5):e10561. doi: 10.1371/journal.pone.0010561 (PMC2866668; doi:10.1371/journal.pone.0010561)

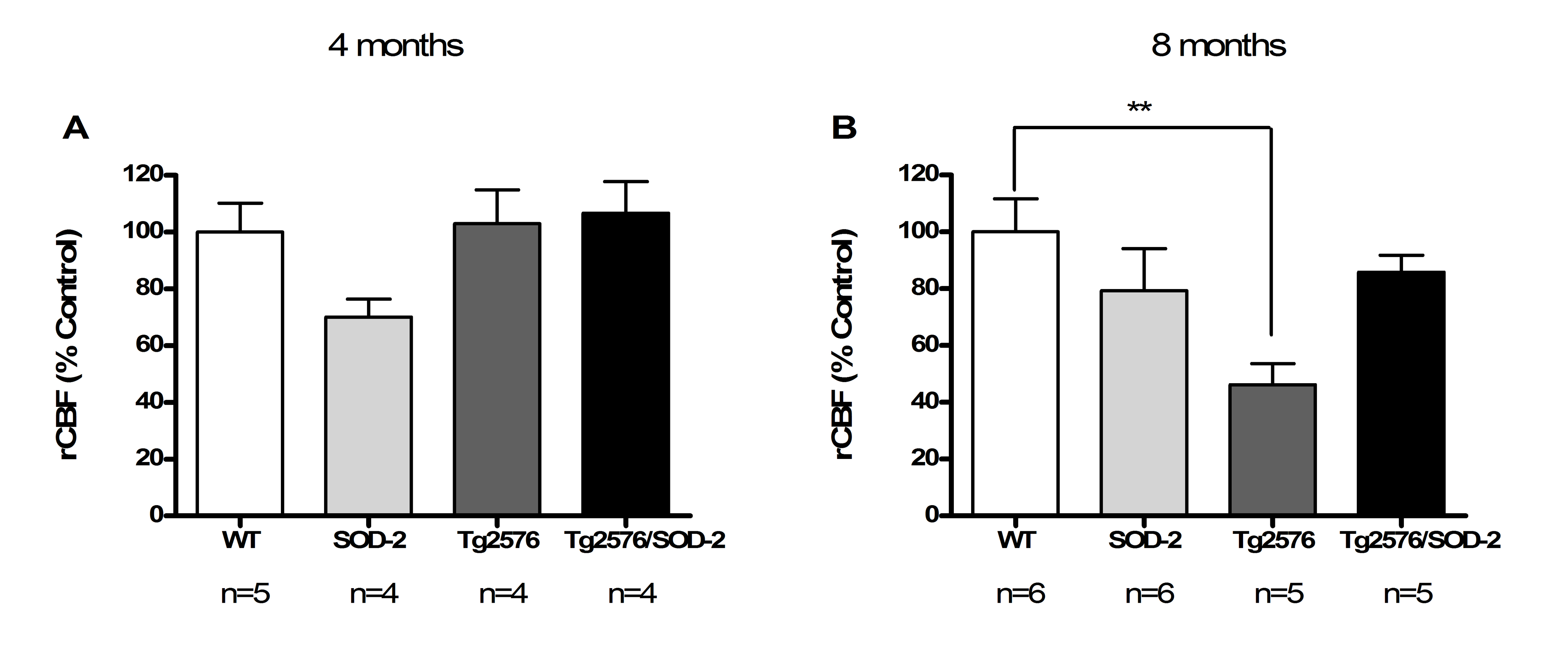

Supplement: Figure S1 — Regional cerebral blood flow measurements at 4 and 8 months of age. A) The graph represents the cerebral blood flow levels in 4 months old WT, SOD-2, Tg2576 and Tg2576/SOD-2 mice as measured by MRI (ASL). No differences in blood flow levels between the different genotypes are observed. B) The graph represents the cerebral blood flow levels in 8 months old WT, SOD-2, Tg2576 and Tg2576/SOD-2 mice as measured by MRI (ASL). Tg2576 mice exhibit a significant deficit in their blood flow that is recovered by SOD-2 overexpression. Significance was assessed by a one-way ANOVA with Dunnett's post-test for multiple comparisons. **p<0.01. (0.87 MB TIF) [file pone.0010561.s001.tif]

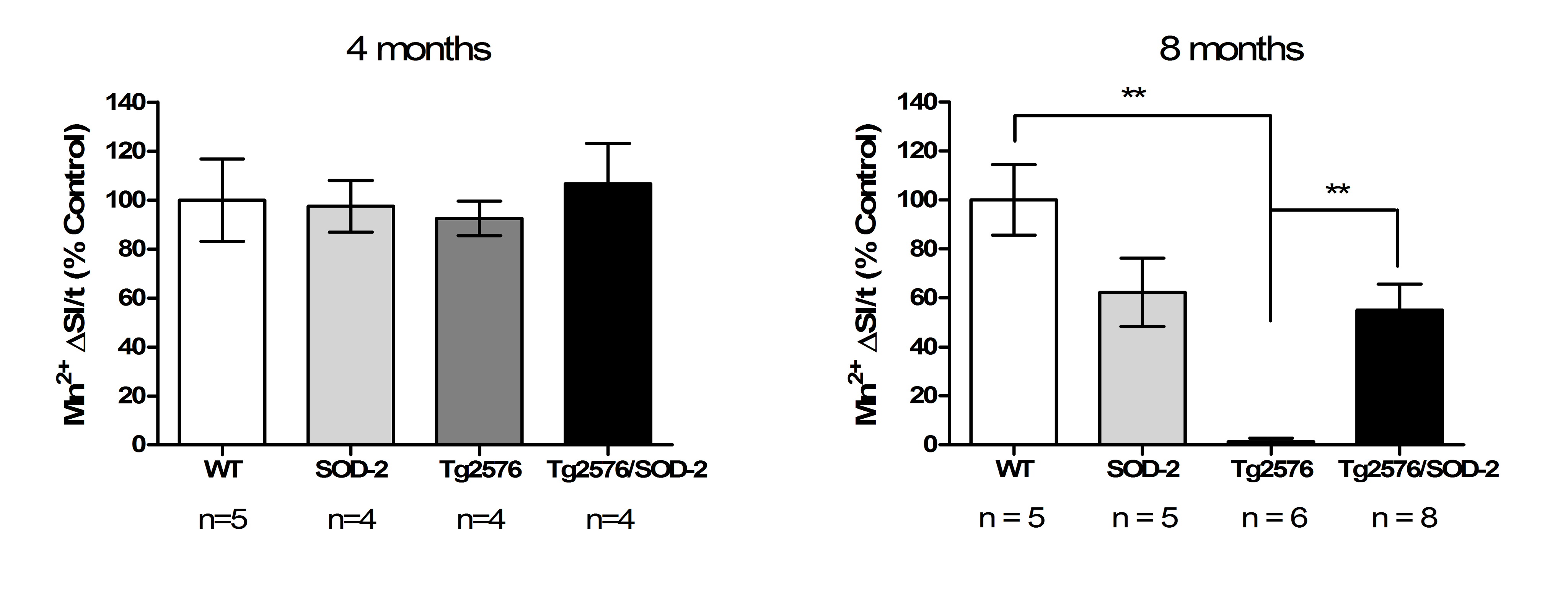

Supplement: Figure S2 — Axonal transport rates measurements at 4 and 8 months of age. A) The graph represents the axonal transport rates measured in vivo by MEMRI in 4 months old WT, SOD-2, Tg2576 and Tg2576/SOD-2 mice. No differences in axonal transport rates between the different genotypes are observed. B) The graph represents the axonal transport rates measured in vivo by MEMRI in 8 months old WT, SOD-2, Tg2576 and Tg2576/SOD-2 mice. Tg2576 mice exhibit a significant deficit in their axonal transport rates that is at least partially recovered by SOD-2 overexpression. Significance was assessed by a one-way ANOVA with Dunnett's post-test for multiple comparisons. **p<0.01. (0.85 MB TIF) [file pone.0010561.s002.tif]

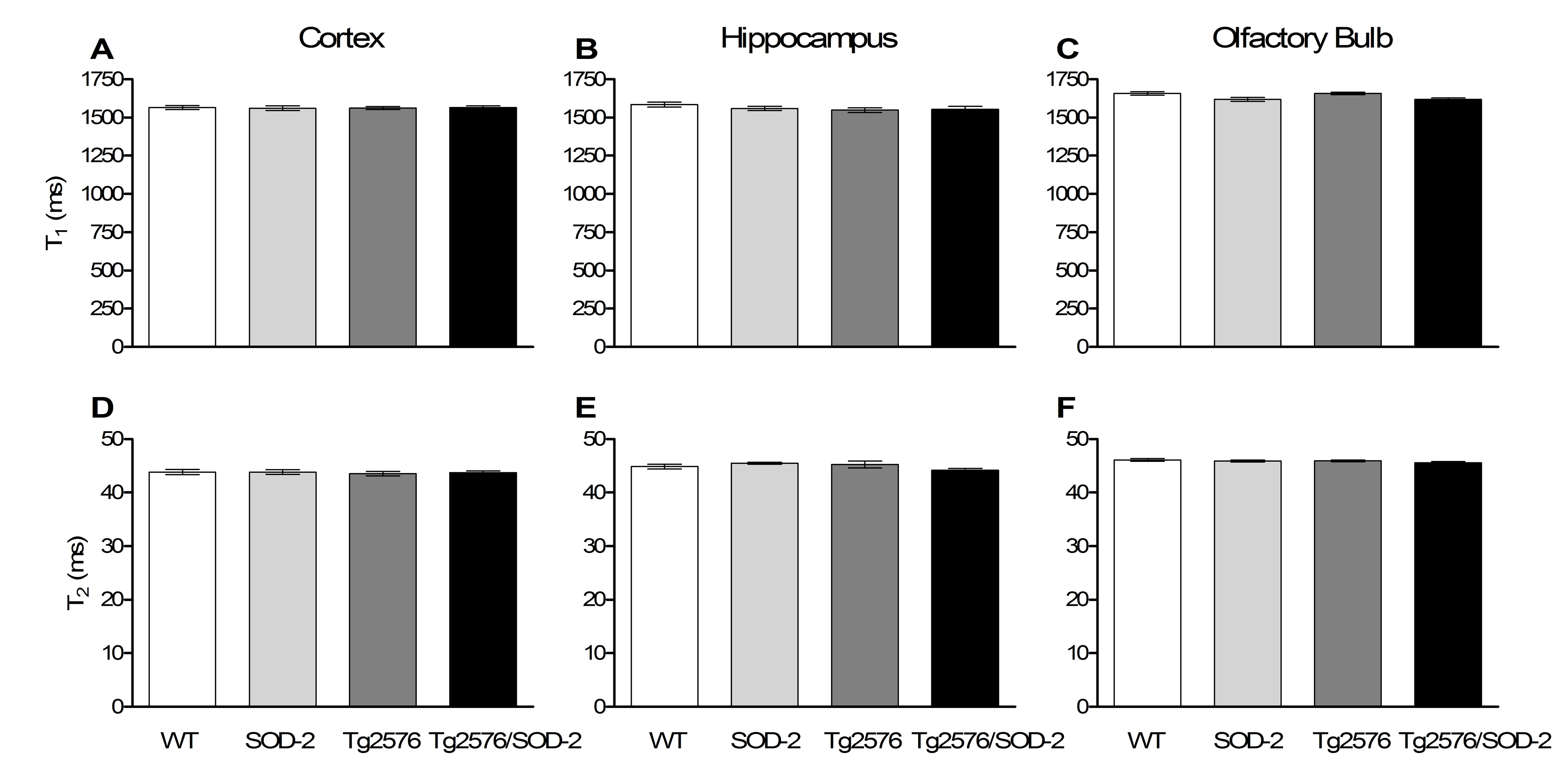

Supplement: Figure S3 — SOD-2 overexpression and the APP-Swedish mutation do not affect the T1 recovery and T2 decay times. A-C) Graphs represent the T1 recovery times measured in the cortex, hippocampus and olfactory bulb of 2 months old WT, SOD-2, Tg2576 and Tg2576/SOD-2 mice. D-F) Graphs represent the T2 decay measured in the cortex, hippocampus and olfactory bulb of 2 month old WT, SOD-2, Tg2576 and Tg2576/SOD-2 mice. (1.35 MB TIF) [file pone.0010561.s003.tif]

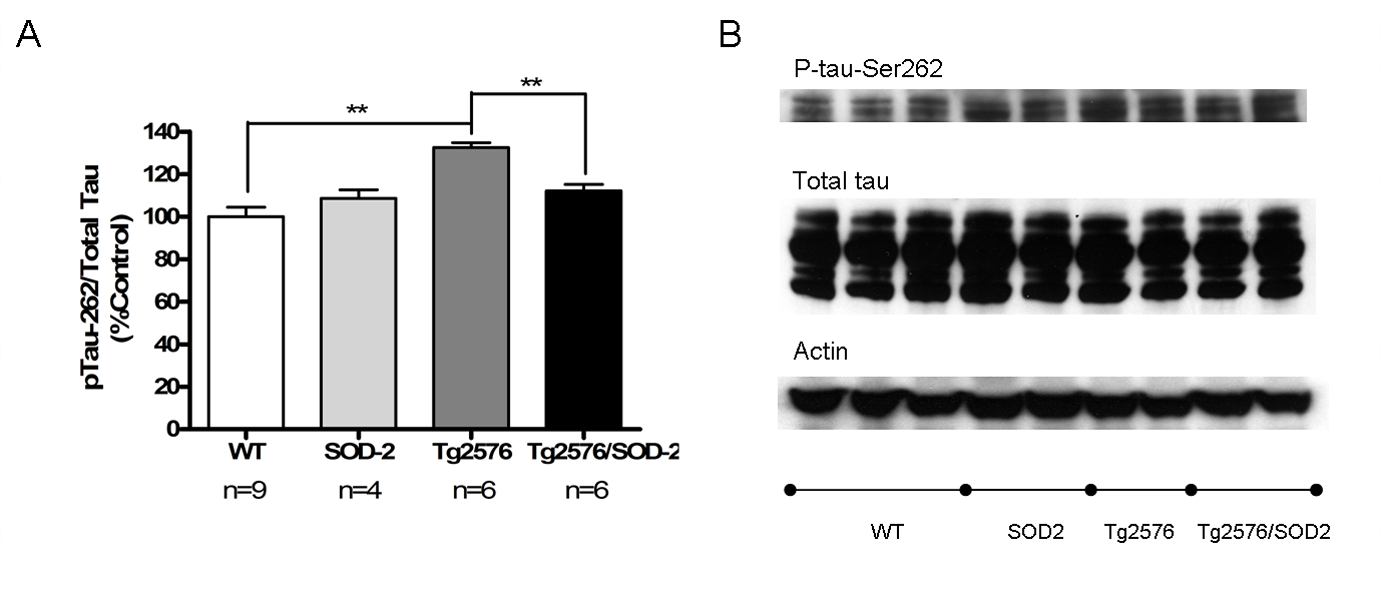

Supplement: Figure S4 — SOD-2 overexpression prevents increases in phospho-tau-ser262 in the brain of Tg2576 mice. A) Graph represents quantification of the levels of phospho-tau-ser262 normalized to total tau from 12 to 16 month old brain homogenates of WT, SOD-2, Tg2576 and Tg2576/SOD-2 mice. Significance was assessed by one way ANOVA with Dunnett's post-test for multiple comparisons. **p<0.01. B) Representative Western blot of phospho-tau-Ser262, total tau and β-actin from brain homogenates of 12 to 16 month old WT, SOD-2, Tg2576 and Tg2576/SOD-2 mice. (0.37 MB TIF) [file pone.0010561.s004.tif]
